# A retrospective analysis of community-onset bloodstream infections at a tertiary-care academic hospital in South Africa. Are current empiric antimicrobial practices appropriate?

**DOI:** 10.1017/ash.2021.236

**Published:** 2021-12-21

**Authors:** Vinitha Alex, Trusha Nana, Vindana Chibabhai

**Affiliations:** 1 Department of Clinical Microbiology and Infectious Diseases, School of Pathology, University of the Witwatersrand, Johannesburg, South Africa; 2 Microbiology Laboratory, Charlotte Maxeke Johannesburg Academic Hospital, National Health Laboratory Services, Johannesburg, South Africa

## Abstract

**Background:**

Community-onset bloodstream infection (CO-BSI) is associated with substantial morbidity and mortality. Knowledge of locally prevalent pathogens and antimicrobial susceptibility patterns can promptly guide appropriate empiric therapy and improve outcomes.

**Objectives:**

We sought to determine the epidemiology of CO-BSI, the blood culture positivity rate and the contamination rate. We also sought to establish appropriateness of current empiric antimicrobial therapy practices.

**Methods:**

We retrospectively analyzed blood cultures taken from January 2015 to December 2019 at the emergency departments (EDs) of a tertiary-care academic hospital in South Africa using extracted laboratory data.

**Results:**

The overall positivity rate of blood cultures taken at the EDs was 15% (95% confidence interval [CI], 0.15–0.16) and the contamination rate was 7% (95% CI, 0.06–0.07). Gram-positive bacteria predominated in the pediatric cohort: neonates, 52 (54%) of 96; infants, 57 (52%) of 109; older children, 63 (61%) of 103. Methicillin-susceptible *Staphylococcus aureus* was the predominant pathogen among older children: 30 (35%) of 85. *Escherichia coli* was the most common pathogen isolated among adults and the elderly: 225 (21%) of 1,060 and 62 (29%) of 214, respectively. Among neonates, the susceptibility of *E. coli* and *Klebsiella pneumoniae* to the combination of ampicillin and gentamicin was 17 (68%) of 25. Among adults, the susceptibility of the 5 most common pathogens to amoxicillin-clavulanate was 426 (78%) of 546 and their susceptibility to ceftriaxone was 481 (85%) of 565 (*P* = .20). The prevalence of methicillin-resistant *S. aureus*, extended-spectrum β-lactamase–producing and carbapenem-resistant Enterobacterales were low among all age groups.

**Conclusions:**

Review of blood culture collection techniques is warranted to reduce the contamination rate. High rates of resistance to currently prescribed empiric antimicrobial agents for CO-BSI warrants a re-evaluation of local guidelines.

Community-onset bloodstream infections (CO-BSIs) are associated with high mortality rates.^1,2^ This situation has been exacerbated by increased antimicrobial resistance (AMR) to commonly prescribed “first-line” antimicrobials. The global increase in community-acquired methicillin-resistant *Staphylococcus aureus* (MRSA), extended-spectrum β-lactamase-producing (ESBL) Enterobacterales and carbapenem-resistant Enterobacterales bloodstream infections (CRE-BSIs) is alarming.^3–5^ Although prompt administration of appropriate antimicrobial agents contributes to improved prognosis in bacteremic patients, the inappropriate use of antimicrobial therapy for BSI is associated with increased morbidity, mortality, and duration of hospitalization and negatively influences healthcare costs.^6,7^ Early identification and rigorous management of sepsis within the first hour dramatically improves patient outcomes.^8^ Determining the epidemiology of CO-BSI can assist in developing effective empiric antimicrobial guidelines that will improve patient outcomes.

The prevalence of CO-BSI, causative pathogens, and antimicrobial susceptibility patterns differ among different age groups, genders, and geographic regions.^1,3,9^ Although the prevalence of clinically significant CO-BSI in Africa has varied widely from 7% to >15%,^1,10^ data for Southern Africa are scarce, particularly in the adult population. A systematic review found that >40% of African countries did not have AMR data.^11^

AMR no longer exists purely as a healthcare-associated entity.^6,12^ It is widespread and increasing at a rate faster than the development of new antimicrobials.^13^ A 2017 estimation study attributed >700,000 deaths globally to AMR, and it is estimated that this will increase to 10 million deaths per year worldwide by 2050.^11,14^

Increasing AMR with increasing age among adults has been described, suggesting the importance of patient age when choosing empiric antimicrobial therapy.^7^ The Standard Treatment Guidelines and Essential Medicines List for South Africa recommends empiric ceftriaxone for suspected sepsis in children with addition of cloxacillin in suspected staphylococcal infections. For neonatal sepsis, the empiric first-line therapy consistent with WHO guidelines is gentamicin combined with ampicillin or benzylpenicillin. Escalation to piperacillin-tazobactam in combination with amikacin is recommended if the child deteriorates on this regimen.^15^ There are no guidelines in South Africa for CO-BSI in adults in the absence of a known source of sepsis. At Charlotte Maxeke Johannesburg Academic Hospital (CMJAH), the empiric choice for CO-BSI at the emergency department (ED) is ampicillin plus gentamicin for neonates and either amoxicillin-clavulanate or ceftriaxone for children and adults, depending on the suspected source of infection. Amoxicillin-clavulanate is used empirically at surgical ED.

The objectives of this study were to determine the positivity and contamination rates of blood cultures taken at the CMJAH EDs and to determine the epidemiology of clinically significant pathogens based on patient age to determine the appropriateness of the current empiric antimicrobial therapy for CO-BSIs.

## Methods

### Study design

We conducted a retrospective laboratory-based study of blood cultures taken at the CMJAH EDs between January 2015 and December 2019. CMJAH is a 1,088-bed tertiary-care academic hospital in Johannesburg that offers numerous specialist services including neonatology, cardiology, pulmonology, infectious diseases, trauma, and oncology.

### Definitions

CO-BSI was defined as a positive blood culture taken from a patient presenting to a CMJAH ED. Organisms isolated from a single blood culture per patient, categorized as contaminants by the US Centers for Disease Control, were regarded as contaminants.^16^ Patients were stratified into 5 age categories: neonates (≤28 days), infants (29 days–1 year), older children (>1–12 years), adults (13–65 years), and elderly (>65 years). Due to the small sample size and wide spectrum of ages, all patients seen at the surgical ED were pooled for analysis.

Multidrug-resistant (MDR) and extensively drug-resistant (XDR) pathogens were classified according to standard definitions described by Magiorakos et al.^17^ These definitions were used exclusively for nonfermenter gram-negative bacteria.

Antimicrobial-resistant pathogens assessed were AmpC and ESBL-producing Enterobacterales, CREs, MDR, and XDR nonfermenter gram-negative bacteria, MRSA, and fluconazole-resistant *Candida* spp.

### Laboratory analysis

The CMJAH microbiology laboratory is a 24-hour laboratory located on the hospital premises with on-site specialist clinical microbiologists. Blood cultures collected from the EDs were incubated in the automated BacT/Alert (bioMerieux, France) incubation system. Organism identification was performed using Vitek MS Matrix assisted laser desorption-ionization-time of flight (MALDI-TOF) or Vitek 2 (bioMerieux, France). Antimicrobial susceptibility testing (AST) was performed using the Kirby-Bauer disc diffusion method, Vitek 2, or gradient diffusion and were interpreted using the Clinical and Laboratory Standards Institute (CLSI M100) standards.^18–22^

### Data analysis

Blood cultures and accompanying microbiology sample data captured on the laboratory information system were analyzed. The data was accessed from the National Health Laboratory Services Corporate Data Warehouse. Positive cultures obtained from the same patient with the same organism within 4 weeks were considered a single episode of CO-BSI.

Microscopy, culture, and susceptibility results of samples including urine, cerebrospinal fluid, pus, fluid, tissue, sputum, tracheal aspirate, and stool taken at the ED were sought to identify the potential source or focus of infection.

Blood culture positivity and contamination rates, epidemiology of clinically significant pathogens causing CO-BSI based on age and prevalence of drug-resistant pathogens were analyzed.

### Statistical analysis

Data were captured in Microsoft 365 Excel (Microsoft, Redmond, WA, 2012). Data are presented as proportions and percentages for individual organisms and antimicrobials. The 2-sample test of proportions was used to calculate the *P* value. Stata/IC version 15.1 statistical software (StataCorp, College Station, TX) was used for analysis. A *P* value < .05 was considered statistically significant.

### Ethical approval

Permission to conduct the study was granted by the Human Research Ethics Committee (Medical) of the University of the Witwatersrand (clearance certificate no. M200341).

## Results

Among 29,286 blood cultures with documented ages collected at CMJAH EDs during the 5-year study period, 23,255 (79%) were collected from adults.

### Positivity and contamination rates

The overall positivity rate of blood culture was 15% (95% confidence interval [CI], 0.15–0.16): pediatric ED (20%; 95% CI, 0.19–0.21), medical ED (14%; 95% CI, 0.13–0.14), and surgical ED (19%; 95% CI, 0.16–0.22).

**Fig. 1.**
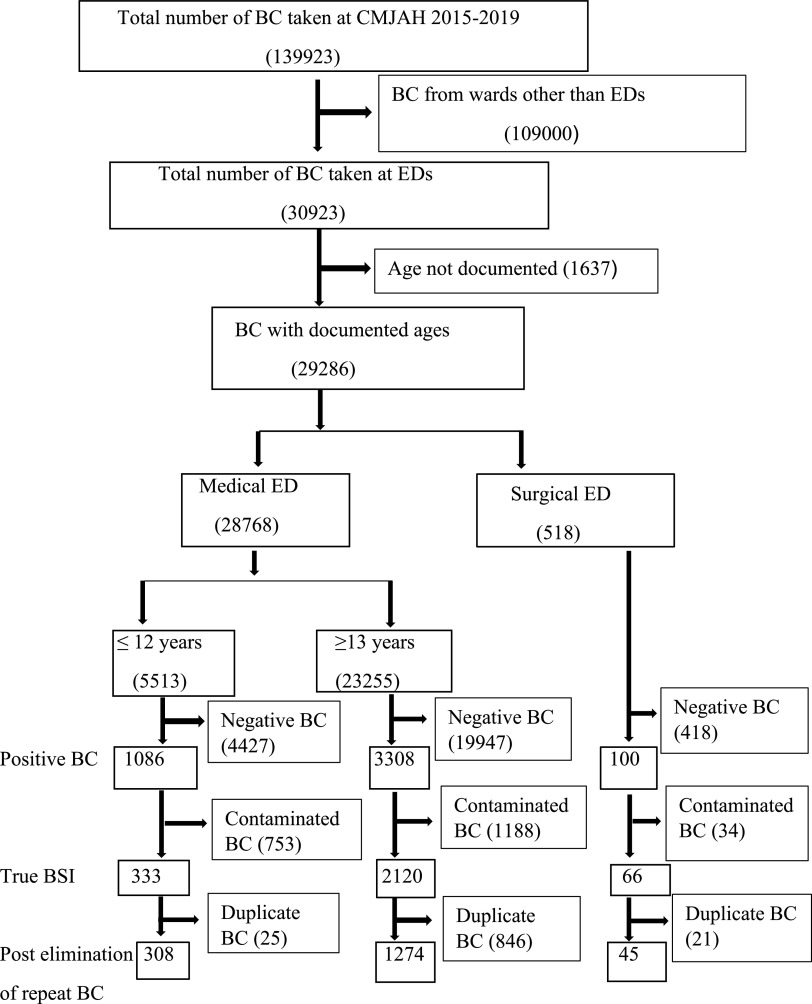
Study flow chart showing blood cultures analysed from the different emergency departments (EDs). Note. BC, blood culture; CMJAH, Charlotte Maxeke Johannesburg Academic Hospital; BSI, bloodstream infection.

The overall contamination rate was 7% (95% CI, 0.06–0.07), with the highest at the pediatric ED (14%; 95% CI, 0.13–0.15) followed by surgical ED (7%; 95% CI, 0.05–0.10) and medical ED (5%; 95% CI 0.04–0.05). Following the exclusion of negative cultures, contaminated cultures, cultures from patients with undocumented age and duplicate cultures, BSIs were identified in 1,274 (6%) of 23,255 patients at the medical ED, in 308 (6%) of 5,513 patients at pediatric ED, and in 45 (9%) of 518 patients at the surgical ED (Fig. 1). The most common contaminant in all EDs was coagulase-negative staphylococci. The distribution of other contaminants varied between different EDs. (Table 1).

**Table 1.** Contaminants

Organism	Pediatric ED, No. (%) [95% CI]	Medical ED, No. (%) [95% CI]	Surgical ED, No. (%) [95% CI]
Coagulase- negative staphylococci	587 (78) [75–81]	829 (70) [67–72]	28 (82) [65–93]
*Viridans streptococci*	90 (12) [9–14]	115 (10) [7–11]	2 (6) [1–19]
*Corynebacterium* spp	29 (4) [3–5]	122 (10) [8–12]	3 (9) [2–23]
Others^a^	47 (6) [4–8]	122 (10) [8–12]	1 (3) [1–15]
Total	753	1,188	34

Note. CI, confidence interval; ED, emergency department.

a *Aerococcus viridans*, *Bacillus* spp other than *Bacillus anthracis, Kocuria* spp, *Lactococcus* spp, *Micrococcus* spp, *Microbacterium spp and Propionibacterium* spp.

### Age-based epidemiology

Among children, gram-positive bacteria predominated across all age groups. Among neonates, 52 (54%) of 96 isolates were gram-positive bacteria. Among infants, 57 (52%) of 109 isolates were gram-positive bacteria, and among older children, 63 (61%) of 103 isolates were gram-positive bacteria (Table 2).

**Table 2.** Rank Order of Pathogens Causing Community-Acquired Bloodstream Infection Among Children

Organism Rank	Pediatric Emergency Department		
	Neonates, No. (%)	Infants, No. (%)	Older Children, No. (%)
1	*Streptococcus agalactiae* 22 (23)	*Escherichia coli, Streptococcus pneumoniae* 19 (17)	*Staphylococcus aureus* 35 (34)
2	*Escherichia coli* 17 (18)	…	*Streptococcus pneumoniae* 18 (17)
3	*Staphylococcus aureus* 16 (17)	*Staphylococcus aureus* 17 (16)	*Bacteroides* spp 7 (7)
4	*Klebsiella* spp 9 (9)	*Haemophilus influenzae, Enterococcus faecalis* 9 (8)	*Escherichia coli, Haemophilus influenzae* 5 (5)
5	*Enterobacter* spp 8 (8)	…	…
6	*Enterococcus faecalis* 7 (7)	*Streptococcus agalactiae* 8 (7)	Non typhoidal *Salmonella* spp 4 (4)
7	Others^a^ 17 (18)	Others^b^ 28 (26)	Others^c^ 29(28)
**Total**	**96**	**109**	**103**

Note. ED, emergency department.

a *Acinetobacter* spp, *Bacteroides* spp, *Candida parapsilosis*, *Enterococcus durans*, *Enterococcus faecium*, *Haemophilus influenzae*, *Listeria monocytogenes*, *Proteus mirabilis*, and *Streptococcus pneumoniae*.

b *Acinetobacter* spp, *Bacteroides* spp, *Campylobacter jejuni*, *Candida albicans*, *Candida lusitaniae, Klebsiella* spp, *Neisseria meningitidis*, *Pantoea* spp, *Proteus* spp, *Pseudomonas oryzihabitans*, *Salmonella* spp, *Streptococcus anginosus*, and *Streptococcus pyogenes*.

c *Acinetobacter* spp, *Campylobacter coli*, *Candida albicans*, *Cryptococcus neoformans*, *Enterococcus cecorum*, *Enterococcus faecalis*, *Enterococcus faecium*, *Klebsiella* spp, *Listeria monocytogenes*, *Moraxella* spp, *N. meningitidis*, *Pseudomonas aeruginosa*, *Salmonella* Typhi, *Stenotrophomonas maltophilia, Streptococcus anginosus*, and *Streptococcus pyogenes*.

A predominance of gram-negative bacteria was noted in adults [507 (48%) of 1,060] and the elderly [148 (69%) of 214]. *E. coli* was the most common pathogen identified in both these groups (Table 3). *C. neoformans* was almost exclusively seen among adults [93 (9%) of 1,060] compared to the elderly, among whom only 1 isolate was identified [1 (0.5%) of 214]. Nonfermenter gram-negative bacteria accounted for 32 (4%) of 907 among adults and 11 (5%) of 201 CO-BSIs among the elderly.

**Table 3.** Rank Order of Pathogens Causing Community-Acquired Bloodstream Infection Among Adults

Organism Rank	Medical ED		Surgical ED, No. (%)
	Adults, No. (%)	Elderly, No. (%)	
1	*Escherichia coli* 225 (21)	*Escherichia coli* 62 (29)	*Staphylococcus aureus* 8 (18)
2	*Streptococcus pneumoniae* 185 (17)	*Klebsiella* spp 28 (13)	*Acinetobacter baumannii, Klebsiella pneumoniae* 7 (16)
3	*Staphylococcus aureus* 159 (15)	*Staphylococcus aureus* 26 (12)	…
4	*Cryptococcus neoformans* 93 (9)	*Enterobacter* spp 13 (6)	*Streptococcus pneumoniae* 6 (13)
5	*Klebsiella* spp 79 (8)	*Streptococcus pneumoniae* 12 (6)	*Escherichia coli, Enterococcus* spp 3 (7)
6	Nontyphoidal *Salmonella* spp 74 (7)	*Proteus* spp 10 (5)	…
7	*Bacteroides* spp 32 (3)	Nontyphoidal *Salmonella* spp 9 (4)	*Proteus* spp, nontyphoidal *Salmonella* spp 2 (4)
8	*Enterococcus faecalis* 22 (2)	*Bacteroides* spp 7 (3)	*…*
9	Others^a^ 191 (18)	Others^b^ 47 (22)	Others^c^ 7 (16)
**Total**	**1060**	**214**	**45**

Note. ED, emergency department.

a *Acinetobacter* spp, *Actinomyces* spp, *Aeromonas hydrophila*, *Alcaligenes* spp, *Burkholderia cepacia*, *Candida albicans*, *Candida glabrata*, *Candida parapsilosis*, *Candida* spp, *Candida tropicalis*, *Citrobacter freundii, Citrobacter koseri*, *Clostridium* spp, *Cryptococcus gattii*, *Enterobacter* spp, *Enterococcus casseliflavus*, *Enterococcus faecium*, *Fusobacterium nucleatum*, *Haemophilus* spp, *Listeria monocytogenes*, *Morganella* spp, *Neisseria meningitidis*, *Paenibacillus lactus*, *Parvimonas micra*, *Plesiomonas shigelloides*, *Prevotella bivia*, *Proteus* spp, *Prototheca*, *Providentia rettgeri*, *Pseudomonas aeruginosa*, *Pseudomonas oryzihabitans*, *Rothia* spp, *Salmonella* Typhi, *Serratia* spp, *Shigella sonnei*, *Streptococcus agalactiae*, *Streptococcus anginosus*, *Streptococcus dysgalactiae*, *Streptococcus gallolyticus*, *Streptococcus pyogenes*, *Staphylococcus lugdunensis*, *Stenotrophomonas maltophilia*, and *Veillonella* spp.

b *Acinetobacter* spp, *Aeromonas hydrophila*, *Burkholderia cepacia*, *Candida albicans*, *Clostridium* spp, *Cryptococcus neoformans*, *Enterococcus faecalis*, *Enterococcus faecium*, *Haemophilus* spp, *Leuconostoc* spp, *Listeria monocytogenes, Moraxella* spp, *Morganella* spp, *Pseudomonas aeruginosa*, *Pseudomonas fluorescens*, *Serratia* spp, *Streptococcus agalactiae*, *Streptococcus anginosus*, *Streptococcus dysgalactiae*, *Streptococcus pyogenes*, and *Staphylococcus lugdunensis*.

c *Candida glabrata*, *Clostridium perfringens*, *Cryptococcus neoformans*, *Enterobacter* spp, *Haemophilus influenzae*, and *Serratia* spp.

*Candida* spp were uncommon across all age groups: 4 (1%) of 308 at the pediatric EDs, 14 (1%) of 1,274 at the adult and elderly EDs and 1 (2%) of 45 at the surgical ED.

### Antimicrobial susceptibility to routinely prescribed antimicrobials

Among neonates, 30 (97%) of 31 gram-positive bacteria (excluding *S. aureus*) were susceptible to ampicillin. Of 16 *S. aureus* isolates, 15 (94%) were susceptible to methicillin. Also, 17 (68%) of 25 *E. coli* and *K. pneumoniae* isolates (the 2 most common gram-negative bacteria identified among neonates) were susceptible to the combination of ampicillin with gentamicin. Of 25 *E. coli* and *K. pneumoniae* isolates, 24 (96%) were susceptible to ampicillin with amikacin (*P* < .05). Among 37 gram-negative isolates overall, 28 (76%) were susceptible to gentamicin. Among 35 gram-negative isolates, 34 (97%) were susceptible to amikacin (*P* < .05). Among the Enterobacterales, 27 (79%) of 34 were susceptible to cefotaxime.

Among infants, all gram-positive bacteria (excluding *S. aureus*) were susceptible to ampicillin. All *S. aureus* isolates were susceptible to cloxacillin. Among Enterobacterales, 21 (75%) of 28 isolates were susceptible to amoxicillin-clavulanate and 28 (85%) of 33 were susceptible to cefotaxime (*P* = .07).

Methicillin-sensitive *S. aureus* (MSSA) contributed to 17% of BSIs among neonates (15 of 89 isolates) and infants (17 of 99 isolates). Among older children, MSSA was isolated in 30 (35%) of 85 CO-BSIs. Among gram-negative bacteria, 4 (57%) of 7 isolates were susceptible to amoxicillin-clavulanate and 13 (87%) of 15 were susceptible to ceftriaxone (*P* < .05).

BSIs due to MSSA were identified in 137 (15%) of 903 adults and 22 (11%) of 201 elderly patients. Among adults, for the 5 most common pathogens, 426 (78%) of 546 isolates were susceptible to amoxicillin-clavulanate and 481 (85%) of 565 were susceptible to ceftriaxone (*P* = 0.2). Among the elderly, for gram-negative bacteria, 70 (65%) of 107 isolates were susceptible to amoxicillin-clavulanate and 88 (80%) of 110 were susceptible to ceftriaxone (*P* < .05). Among 130 gram-negative bacteria isolates, 108 (83%) were susceptible to piperacillin-tazobactam.

At the surgical ED, among gram-negative bacteria, 10 (59%) of 17 isolates were susceptible to amoxicillin-clavulanate and 12 (75%) of 16 were susceptible to ceftriaxone (*P* < .05). Among 22 gram-negative isolates, 16 (73%) were susceptible to piperacillin-tazobactam in this group. All *S. aureus* identified in this unit were susceptible to cloxacillin.

Among 13 *Candida* spp isolated from all EDs, 12 (92%) were susceptible to fluconazole. The only resistant isolate was a *Candida parapsilosis*, which was isolated from a neonate. Detailed AST data are available in the Supplementary Material.

### Prevalence of multidrug-resistant pathogens

MRSA was an uncommon cause of BSI across all age groups: 1 (1%) of 89 neonates, 3 (3%) of 87 older children, 11 (1%) of 903 adults, and 4 (2%) of 201 elderly patients (Table 4).

**Table 4.** Resistance Profile of Selected Pathogens

Resistance	Neonates No./Total (%)	Infants No./Total (%)	Older Children No./Total (%)	Adults No./Total (%)	Elderly No./Total (%)	Surgical ED No./Total (%)
**Gram-positive bacteria**	49/89 (55)	56/99 (57)	60/87 (69)	414/907 (46)	60/201 (30)	18/45 (40)
*Enterococcus* spp	10/49 (20)	9/56 (16)	5/60 (8)	25/414 (6)	8/60 (13)	4/18 (22)
VRE	0/10 (0)	0/9 (0)	0/4 (0)	0/23 (0)	0/8 (0)	0/4 (0)
*Staphylococcus aureus*	16/49 (33)	17/56 (30)	33/60 (55)	152/414 (37)	26/60 (43)	8/18 (44)
MRSA	1/16 (6)	0/17 (0)	3/33 (9)	11/148 (7)	4/26 (15)	0/8 (0)
**Gram-negative bacteria**	39/89 (44)	41/99 (41)	24/87 (28)	459/907 (51)	139/201 (69)	26/45 (58)
Enterobacterales	35/39 (90)	29/41 (70)	14/24 (58)	417/459 (91)	124/139 (89)	18/26 (69)
AmpC	8/35 (23)	0/29 (0)	0/14 (0)	20/417 (5)	12/124 (10)	2/18 (11)
ESBL	6/35 (17)	5/29 (17)	1/14 (7)	61/417 (15)	19/124 (15)	4/18 (22)
CRE	1/35 (3)	0/29 (0)	1/14 (7)	11/417 (3)	6/124 (5)	1/18 (6)
Nonfermenters	3/39 (8)	3/41 (7)	6/24 (25)	32/459 (7)	11/139 (8)	7/26 (27)
MDR	0/3 (0)	0/3 (0)	0/6 (0)	1/32 (3)	0/11 (0)	4/7 (57)
XDR	0/3 (0)	0/3 (0)	0/6 (0)	4/32 (13)	2/11 (18)	1/7 (14)

Note. ED, emergency department; VRE, vancomycin-resistant enterococci; MRSA, methicillin-resistant *Staphylococcus aureus;* ESBL, extended-spectrum β-lactamase; CRE, carbapenem-resistant Enterobacterales; MDR, multidrug resistant; XDR, extensively drug resistant.

AmpC-producing Enterobacterales accounted for BSIs among 8 (9%) of 89 neonates, 20 (5%) of 414 adults, 12 (6%) of 201 elderly patients, and 2 (4%) of 45 surgical patients.

ESBL-producing Enterobacterales were identified from all EDs: 6 (7%) of 89 neonates, 5 (5%) of 99 infants, 1 (1%) of 87 older children, 64 (7%) of 907 adults, and 19 (9%) of 201 elderly patients. The prevalence of ESBL-producing Enterobacterales in the surgical ED was 4 (9%) of 45.

CREs were identified in 1% of BSIs among neonates (1 of 89), older children (1 of 87), and adults (11 of 907). Among patients seen at the surgical ED, the prevalence was 1 (2%) of 45, and among the elderly the prevalence was 6 (3%) of 201.

XDR non-fermenter gram-negative bacteria were identified in 3 (0.3%) of 907 adults, 2 (0.9%) of 201 elderly patients, and 1 (2%) of 45 BSIs among surgical ED patients (Table 4).

### Analysis of potential source/focus of sepsis

Among neonates, 66 (75%) of 88 blood cultures were accompanied by cultures from other sites. A potential focus or source of sepsis was identified in 27 (41%) of 66 BSIs. Meningitis was the most common focus of infection [14 (52%) of 27] followed by urinary tract infections [UTIs; 8 (30%) of 27]. Meningitis was also the most common focus of infection among infants [10 (26%) of 39] and older children [5 (17%) of 29].

At the surgical ED, among the blood cultures with concomitant cultures from other sites [17 (38%) of 45], the potential source of infection was identified in 10 (22%) of 45 BSIs. UTIs were the predominant source of infection in 4 (40%) of 10 cases. Among adults, 355 (39%) of 906 blood cultures were accompanied by cultures from other sites, and among the elderly, 80 (40%) of 201 blood cultures were accompanied by cultures from other sites. UTIs were the most common source of infection, with 57 (16%) of 355 cases among adults and 25 (31%) of 80 cases among the elderly.

## Discussion

This is the first study describing the epidemiology of CO-BSIs at CMJAH EDs. The positivity rates in our study ranged between 14% and 20% and were higher than those described in other studies.^23^ Contamination rates in our study (ie, 5%–14%) were higher than the internationally acceptable standard of 2%–3%.^24^ Gram-positive bacteria were the predominant pathogens in our pediatric cohort. Gram-negative bacteria dominated among adults and the elderly. *E. coli* was the most prevalent gram-negative pathogen across all age groups. Despite *S. aureus* showing preponderance across all age groups, the prevalence of MRSA was low. Although the prevalence of ESBL-producing Enterobacterales and CREs was low, high rates of resistance of gram-negative bacteria to currently prescribed empiric antibiotics were noted among neonates, adults, and the elderly.

Limiting collection of blood cultures to patients with a high likelihood of BSI and adherence to blood culture collection guidelines can assist in decreasing contamination rates.^25,26^ The possible reason for the higher positivity rate in this study could be that the patients who present to this institution usually have a greater severity of illness and are often bacteremic. The high volume of patients and inadequate resources possibly results in suboptimal aseptic techniques, thus increasing the contamination rate.

In our study, the majority of pediatric BSIs occurred among infants. As reported in a South African study that reviewed community-acquired BSI in children aged 0–14 years, gram-positive bacteria predominated in our pediatric cohort, too.^12^ More than half the isolates responsible for BSI among children of all age groups were gram-positive bacteria. Similar to other studies, *S. aureus* was a predominant pathogen across all age groups in our study.^4,12,27,28^ *S. pneumoniae,* reported as the most common pathogen associated with CO-BSI among African children,^2^ was prominent among infants and older children. South African surveillance data have shown that invasive pneumococcal disease (IPD) in children aged <5 years is predominantly due to nonvaccine serotypes, whereas in older individuals it includes both vaccine and nonvaccine serotypes.^29^ *S. pneumoniae* BSI was higher among adults than among the elderly, which could be related to the higher incidence of HIV among adults.^30^ Children and adults at risk for IPD should be adequately vaccinated to protect against severe disease. Although *S. agalactiae* was predominantly seen among neonates, it was also identified among infants, adults, and the elderly. This finding contrasts with those of other studies in which it was identified exclusively in neonates and adults aged >60 years.^9^

Enterobacterales were the commonest group of gram-negative pathogens with *E. coli* predominating across all age groups, consistent with the findings of several studies.^12,27,28^ The most prevalent isolate in CO-BSI among adults in Africa, nontyphoidal *Salmonella*,^2^ was uncommon in our study, similar to another study from South Africa.^12^ This difference is likely due to the improved management of HIV in South Africa, a known risk factor for nontyphoidal *Salmonella* infections. The high prevalence of HIV (19%) among adults in South Africa, a well-established predisposing factor for invasive cryptococcal disease, could be the reason for the high rate of recovery of *C. neoformans* from BSI in this group.^31,32^

Although gram-positive bacteria (excluding *S. aureus*) had good susceptibility rates to ampicillin among neonates, the susceptibility of the most common gram-negative bacteria to the current empiric therapy of ampicillin in combination with gentamicin was low (68%). For this reason and the high prevalence of MSSA (17%), cefotaxime monotherapy is recommended with the addition of amikacin to be considered in neonates with high risk for MDR pathogens. This risk includes prior exposure to healthcare or antibiotics, particularly in very low birth-weight and premature neonates.

The high prevalence of MSSA in older children supports the current South African empiric therapy guideline of ceftriaxone. Treatment should be changed to cloxacillin if culture confirms *S. aureus* BSI. When meningitis is not the reason for admission, amoxicillin- clavulanate is a suitable alternative.

In this study, gram-negative bacteria were the predominant cause of BSI among adults and elderly patients. Ceftriaxone is recommended for the empiric treatment of CO-BSI among adults. Consistent with previous reports, increasing AMR with increasing age was evident in our study.^7^ The elderly had higher proportions of AmpC, ESBL-, and CRE-producing Enterobacterales compared to adults and children. This finding could be related to increased comorbidities, exposure to health care, and antimicrobial use among the elderly. The addition of amikacin to ceftriaxone as a second agent is recommended in this population due to the high risk of MDR pathogens.

To provide a conclusive recommendation on empiric therapy for surgical patients, further studies focusing on this cohort with additional clinical information is required.

Although MRSA was isolated in several age groups, the prevalence was lower (0%–3%) than that described in other studies, in which it varied from 6% to 37%.^5,9,28^ Empiric antibacterial cover for MRSA is not recommended in this population.

The prevalence of ESBL-producing Enterobacterales showed wide variation (1%–9%) between the different EDs, with the highest prevalence in our study surpassing levels of 7% to 8% described elsewhere.^6,9^

The potential source of infection was not identified for most BSIs. Although cultures from sites other than blood were collected in 75% of neonatal cases, <40% of BSIs had concomitant cultures from the other EDs. Meningitis was the most common focus among children, and UTI was the most frequently identified source among adult, elderly, and surgical patients. Identification of the focus of infection is important to guide empiric antimicrobial therapy because the prescribed drugs must achieve adequate tissue concentrations at the site of infection.^33^ Efforts should be made to submit samples from EDs for microbiological investigation from the most likely source of sepsis prior to the initiation of antibiotics. Due to the frequent presentation of nonspecific symptoms in neonates, identification of a source can be challenging. Cultures from cerebrospinal fluid and urine are advisable in the absence of an obvious source in this group. However, the collection of samples from other sites should not delay the initiation of antibiotics in critically ill patients with sepsis or septic shock.^33^

The findings of this study provide evidence for continued pathogen surveillance at hospital EDs, and they support the need for current guideline revision for empiric treatment of CO-BSI among adults and children.

This study had several limitations. It was conducted in a single tertiary-care center; the findings may not be universally applicable. Due to the retrospective nature of the study, the current epidemiology and AMR might not be adequately reflected. Although all blood cultures analyzed were collected in the EDs, recent contact with other healthcare facilities and other risk factors for MDR pathogens could not be determined; thus, healthcare associated infections could have been inadvertently included. Adequacy of blood culture collection techniques, such as collection prior to initiation of antimicrobial therapy and volume of blood collected, both of which could lead to underestimation of BSI, could not be ascertained. Patients charts which could provide additional information regarding source of infection and predisposing factors for BSI (diabetes, immunosuppression, etc) were not reviewed. Lastly, the outcomes associated with empiric therapy were not available. The recommendations made above should thus be applied judiciously based on the clinical context.

Ongoing local and national surveillance of the epidemiology and AMR in CO-BSI is recommended to ensure that guidelines for empiric therapy remain appropriate. Follow-up studies are needed to determine whether implementation of these recommendations affects the clinical outcomes of patients with CO-BSI.

## Supplementary material

For supplementary material accompanying this paper visit https://doi.org/10.1017/ash.2021.236. click here to view supplementary material
