# Supplementary material for: A retrospective analysis of community-onset bloodstream infections at a tertiary-care academic hospital in South Africa. Are current empiric antimicrobial practices appropriate?
Source: Antimicrob Steward Healthc Epidemiol. 2021 Dec 21;1(1):e67. doi: 10.1017/ash.2021.236 (PMC9495636; doi:10.1017/ash.2021.236)
Supplement: Supplementary file 1 [file S2732494X21002369sup001.pdf]

Supplementary tables

Supplementary Table 1: Gram- negative AST results for neonates

Serial number	Organisms	Number of isolates (duplicates removed)	Breakdown of each isolate	% Susceptibility to Routinely Tested Antimicrobial Agents													% Resistance			
				Beta Lactams										Aminoglycosides						
				Ampicillin	Augmentin	Pip-taz	Ceftiaxone	Ceftazidime	Cefepime	Ertapenem	Meropenem	Imipenem	Ciprofloxacin	Getamicin	Amikacin	Cotrimoxazole	% ESBL	% CRE	% MDR	% XDR
1	*Escherichia coli*	16	Total no. isolates with sensitivities	16	16	16	16	16	16	16	15	16	15	16	16	16	19%	0%	_	_
			No. of susceptible isolates	4	13	16	13	15	15	16	15	16	13	13	16	5				
			% Susceptible Isolates	25%	81%	100%	81%	94%	94%	100%	100%	100%	87%	81%	100%	31%				
2	*Klebsiella pneumoniae*	9	Total no. isolates with sensitivities	_	9	9	9	8	9	8	8	9	8	9	9	8	33%	11%	_	_
			No. of susceptible isolates	_	4	5	5	5	5	8	7	8	8	4	8	5				
			% Susceptible Isolates	_	44%	56%	56%	63%	56%	100%*	88%	89%	100%	44%	89%	63%				
3	*Enterobacter spp.*	8	Total no. isolates with sensitivities	_	_	7	7	7	8	7	7	7	6	7	7	7	0%	0%	_	_
			No. of susceptible isolates	_	_	7	7	7	8	7	7	7	6	7	7	6				
			% Susceptible Isolates	_	_	100%	100%	100%	100%	100%	100%	100%	100%	100%	100%	86%				
4	*Acinetobacter spp.*	3	Total no. isolates with sensitivities	_	_	3	_	3	3	_	3	3	3	3	1	3	_	_	0%	0%
			No. of susceptible isolates	_	_	3	_	3	3	_	3	3	3	3	1	3				
			% Susceptible Isolates			100%		100%	100%	_	100%	100%	100%	100%	100%	100%				
5	*Proteus* spp.	2	Total no. isolates with sensitivities	2	2	2	2	2	2	2	0	1	0	2	2	2	0%	0%	_	_
			No. of susceptible isolates	1	2	2	2	2	2	2	_	1	_	1	2	1				
			% Susceptible Isolates	50%	100%	100%	100%	100%	100%	100%	_	100%	_	50%	100%	50%				
6	*Haemophilus influenzae*	1	Total no. isolates with sensitivities	1	0	0	0	_	0	0	1	_	1	_	_	1	_	_	_	_
			No. of susceptible isolates	1	_	_	_	_	_	_	1	_	1	_	_	0				
			% Susceptible Isolates	100%	_	_	_	_	_	_	100%	_	100%	_	_	0%				

* fewer susceptibility results were available for ertapenem, hence the lowest susceptibility result for a carbapenem was used to determine the CRE rate.

AST=antimicrobial susceptibility testing; spp.= species; No.= number; Pip-taz= piperacillin-tazobactam; ESBL= extended spectrum beta lactamase; CRE= carbapenem resistant Enterobacterales; MDR= multidrug resistant; XDR= extensively drug resistant

Supplementary Table 2: Gram- positive AST results for neonates

Serial number	Organisms	Number of isolates (duplicates removed)	Breakdown of each isolate	% Susceptibility to Routinely Tested Antimicrobial Agents									Resistance	
				Cloxacillin	Penicillin/ Ampicillin	Clindamycin	Vancomycin	Linezolid	Cotrimoxazole	Rifampicin	Gentamicin high	Ciprofloxacin	% MRSA	% VRE
1	***Streptococcus agalactiae***	**21**	**Total no. isolates with sensitivities**	_	20	_	19	19	_	_	_	_	_	_
			**No. of susceptible isolates**	_	20	_	19	18	_	_	_	_		
			**% Susceptible Isolates**	_	**100%**	_	**100%**	**95%**	_					
2	***Staphylococcus aureus***	**16**	**Total no. isolates with sensitivities**	16	_	15	16	15	15	16	_	15	**6%**	_
			**No. of susceptible isolates**	15	_	13	16	15	8	16	_	14		
			**% Susceptible Isolates**	**94%**	_	**87%**	**100%**	**100%**	**53%**	**100%**		**93%**		
3	***Enterococcus spp.***	**10**	**Total no. isolates with sensitivities**	_	10	_	10	9	_	_	10	_	_	**0%**
			**No. of susceptible isolates**	_	9	_	10	9	_	_	7	_		
			**% Susceptible Isolates**	_	**90%**	_	**100%**	**100%**	_		**70%**			
4	***Streptococcus pneumoniae***	**2**	**Total no. isolates with sensitivities**	_	1	_	2	2	_	_	_	_	_	_
			**No. of susceptible isolates**	_	1	_	2	2	_	_		_		
			**% Susceptible Isolates**	_	**100%**	_	**100%**	**100%**	_	_	_	_		

AST= antimicrobial susceptibility testing; spp.= species; No.= number; MRSA= methicillin resistant *Staphylococcus aureus*; VRE= vancomycin resistant *Enterococcus*

Supplementary Table 3: Gram- negative AST results for infants

Serial number	Organisms	Number of isolates (duplicates removed)	Breakdown of each isolate	% Susceptibility to Routinely Tested Antimicrobial Agents													% Resistance			
				Beta Lactams										Aminoglycosides						
				Ampicillin	Augmentin	Pip-taz	Ceftiaxone	Ceftazidime	Cefepime	Ertapenem	Meropenem	Imipenem	Ciprofloxacin	Getamicin	Amikacin	Cotrimoxazole	% ESBL	% CRE	% MDR	% XDR
1	*Escherichia coli*	19	Total no. isolates with sensitivities	18	19	19	19	19	18	17	17	18	15	17	19	19				
			No. of susceptible isolates	4	15	19	17	18	18	17	17	18	14	15	19	7	11%	0%	_	_
			% Susceptible Isolates	22%	79%	100%	89%	95%	100%	100%	100%	100%	93%	88%	100%	37%				
2	*Haemophilus influenzae*	8	Total no. isolates with sensitivities	8	0	0	4	_	0	0	7	_	3	_	_	7				
			No. of susceptible isolates	5	_	_	4	_	_	_	7	_	3	_	_	4	_	_	_	_
			% Susceptible Isolates	63%	_	_	100%	_	_	_	100%	_	100%	_	_	57%				
3	*Klebsiella spp.*	7	Total no. isolates with sensitivities	_	7	7	7	6	7	7	7	7	7	7	6	7				
			No. of susceptible isolates	_	4	4	4	4	4	7	7	7	6	4	6	3	43%	0%	_	_
			% Susceptible Isolates	_	57%	57%	57%	67%	57%	100%	100%	100%	86%	57%	100%	43%				
4	*Acinetobacter spp.*	3	Total no. isolates with sensitivities	_	_	3	_	3	3	_	3	2	3	2	2	3				
			No. of susceptible isolates	_	_	3	_	3	3	_	3	2	3	2	2	3	_	_	0%	0%
			% Susceptible Isolates			100%		100%	100%		100%	100%	100%	100%	100%	100%				
5	*Pantoea spp.*	2	Total no. isolates with sensitivities	2	1	1	2	0	1	1	0	1	1	1	0	0				
			No. of susceptible isolates	0	1	1	2	_	1	1	_	1	1	1	_	_	0%	0%	_	_
			% Susceptible Isolates	0%	100%	100%	100%	_	100%	100%	_	100%	100%	100%	_	_				
6	*Salmonella* spp.	1	Total no. isolates with sensitivities	1	_	_	1	1	_	1	_	_	1	_	_	1				
			No. of susceptible isolates	1	_	_	1	1	_	1	_	_	1	_	_	1	0%	0%	_	_
			% Susceptible Isolates	33%	_	_	100%	100%	_	100%	_	_	100%	_	_	100%				
7	*Campylobacter jejuni*	1	Total no. isolates with sensitivities	_	_	_	_	_	_	_	_	_	1	_	_	_				
			No. of susceptible isolates	_	_	_	_	_	_	_	_	_	1	_	_	_	_	_	_	_
			% Susceptible Isolates	_	_	_	_	_	_	_	_	_	100%	_	_	_				

AST=antimicrobial susceptibility testing; spp.= species; No.= number; Pip-taz= piperacillin-tazobactam; ESBL= extended spectrum beta lactamase; CRE= carbapenem resistant Enterobacterales; MDR= multidrug resistant; XDR= extensively drug resistant

Supplementary Table 4: Gram- positive AST results for infants

Serial number	Organisms	Number of isolates (duplicates removed)	Breakdown of each isolate	% Susceptibility to Routinely Tested Antimicrobial Agents										Resistance	
				Cloxacillin	Penicillin/ Ampicillin	Clindamycin	Vancomycin	Linezolid	Cotrimoxazole	Rifampicin	Gentamicin higl	Ciprofloxacin	Ceftriaxone	% MRSA	% VRE
1	*Streptococcus pneumoniae*	19	Total no. isolates with sensitivities	_	1	_	19	18	_	_	_	_	2	_	_
			No. of susceptible isolates	_	1	_	19	18	_	_	_	_	2		
			% Susceptible Isolates	_	100%	_	100%	100%	_	_	_	_	100%		
2	*Staphylococcus aureus*	17	Total no. isolates with sensitivities	17	_	17	17	16	16	17	_	16	_	0%	_
			No. of susceptible isolates	17	_	16	17	16	9	17	_	13	_		
			% Susceptible Isolates	100%	_	94%	100%	100%	56%	100%	_	81%	_		
3	*Enterococcus faecalis*	9	Total no. isolates with sensitivities	_	9	_	9	9	_	_	8	_	_	_	0%
			No. of susceptible isolates	_	9	_	9	9	_	_	5	_	_		
			% Susceptible Isolates	_	100%	_	100%	100%	_	_	63%	_	_		
4	*Streptococcus agalactiae*	8	Total no. isolates with sensitivities	_	6	_	8	8	_	_	_	_	_	_	_
			No. of susceptible isolates	_	6	_	8	8	_	_	_	_	_		
			% Susceptible Isolates	_	100%	_	100%	100%	_	_	_	_	_		
5	*Other Streptococci (S.pyogenes,S. anginosus)*	3	Total no. isolates with sensitivities	_	2	_	3	3	_	_	_	_	3	_	_
			No. of susceptible isolates	_	2	_	3	3	_	_	_	_	3		
			% Susceptible Isolates	_	100%	_	100%	100%	_	_	_	_	100%		

AST= antimicrobial susceptibility testing; spp.= species; No.= number; MRSA= methicillin resistant *Staphylococcus aureus*; VRE= vancomycin resistant *Enterococcus*

Supplementary Table 5: Gram- negative AST results for older children

Serial number	Organisms	Number of isolates (duplicates removed)	Breakdown of each isolate	% Susceptibility to Routinely Tested Antimicrobial Agents													% Resistance			
				Beta Lactams										Aminoglycosides						
				Ampicillin	Augmentin	Pip-taz	Ceftiaxone	Ceftazidime	Cefepime	Ertapenem	Meropenem	Imipenem	Ciprofloxacin	Getamicin	Amikacin	Cotrimoxazole	% ESBL	% CRE	% MDR	% XDR
			**Total no. isolates with sensitivities**	5	5	5	5	5	5	5	5	5	5	5	5	5				
**1**	***Escherichia coli***	**5**	**No. of susceptible isolates**	0	4	4	5	5	5	5	5	5	4	5	5	0	**0%**	**0%**	_	_
			**% Susceptible Isolates**	**0%**	**80%**	**80%**	**100%**	**100%**	**100%**	**100%**	**100%**	**100%**	**80%**	**100%**	**100%**	**0%**				
			**Total no. isolates with sensitivities**	4	0	0	2	_	)	0	5	_	0	_	_	4				
**2**	***Haemophilus influenzae***	**5**	**No. of susceptible isolates**	2	_	_	2	_	_	_	5	_	_	_	_	1	_	_	_	_
			**% Susceptible Isolates**	**50%**	_	_	**100%**	_	_	_	**100%**	_	_	_	_	**25%**				
			**Total no. isolates with sensitivities**	_	2	2	2	2	2	1	2	2	2	2	2	2				
**3**	***Klebsiella spp.***	**2**	**No. of susceptible isolates**	_	0	0	0	0	0	1	1	1	2	0	1	0	**50%**	**50%**	_	_
			**% Susceptible Isolates**	_	**0%**	**0%**	**0%**	**0%**	**0%**	**100%***	**50%**	**50%**	**100%**	**0%**	**50%**	**0%**				
			**Total no. isolates with sensitivities**	_	_	1	_	2	2	_	2	2	1	2	2	2				
**4**	***Acinetobacter spp.***	**2**	**No. of susceptible isolates**	_	_	1	_	1	1	_	2	2	1	2	2	2	_	_	**0%**	**0%**
			**% Susceptible Isolates**	_	_	**100%**	_	**50%**	**50%**	_	**100%**	**100%**	**100%**	**100%**	**100%**	**100%**				
			**Total no. isolates with sensitivities**	_	_	2	_	2	2	_	2	2	2	2	2	_				
**5**	***Pseudomonas aeruginosa***	**2**	**No. of susceptible isolates**	_	_	2	_	2	2	_	2	2	2	2	2	_	_	_	**0%**	**0%**
			**% Susceptible Isolates**	_	_	**100%**	_	**100%**	**100%**	_	**100%**	**100%**	**100%**	**100%**	**100%**	_				
			**Total no. isolates with sensitivities**	7	_	_	6	3	_	7	_	_	7	_	_	6				
**6**	***Salmonella* spp. and *S. typhi***	**7**	**No. of susceptible isolates**	3	_	_	6	3	_	7	_	_	4	_	_	3	**0%**	**0%**	_	_
			**% Susceptible Isolates**	**43%**	_	_	**100%**	**100%**	_	**100%**	_	_	**57%**	_	_	**50%**				

*fewer susceptibility results were available for ertapenem, hence the lowest susceptibility result for a carbapenem was used to determine the CRE rate.

AST=antimicrobial susceptibility testing; spp.= species; No.= number; Pip-taz= piperacillin-tazobactam; ESBL= extended spectrum beta lactamase; CRE= carbapenem resistant Enterobacterales; MDR= multidrug resistant; XDR= extensively drug resistant

Supplementary Table 6: Gram- positive AST results for older children

Serial number	Organisms	Number of isolates (duplicates removed)	Breakdown of each isolate	% Susceptibility to Routinely Tested Antimicrobial Agents										Resistance	
				Cloxacillin	Penicillin/ Ampicillin	Clindamycin	Vancomycin	Linezolid	Cotrimoxazole	Rifampicin	Gentamicin hig	Ciprofloxacin	Ceftriaxone	% MRSA	% VRE
**1**	***Staphylococcus aureus***	**35**	**Total no. isolates with sensitivities**	33	_	33	34	35	35	35	_	34	_	9,0%	_
			**No. of susceptible isolates**	30	_	29	34	35	14	33	_	30	_		
			**% Susceptible Isolates**	**91%**	_	**88%**	**100%**	**100%**	**40%**	**94%**	_	**88%**	_		
**2**	***Streptococcus pneumoni***	**17**	**Total no. isolates with sensitivities**	_	5	_	17	16	_	_	_	_	4	_	_
			**No. of susceptible isolates**	_	4	_	17	16	_	_	_	_	4		
			**% Susceptible Isolates**	_	**80%**	_	**100%**	**100%**	_	_	_	_	**100%**		
**3**	***Enterococcus* spp.**	**5**	**Total no. isolates with sensitivities**	_	4	_	4	4	_	_	4	_	_	_	**0%**
			**No. of susceptible isolates**	_	1	_	4	4	_	_	2	_	_		
			**% Susceptible Isolates**	_	**25%**	_	**100%**	**100%**	_	_	**50%**	_	_		
**4**	***Other Streptococci (S.pyogenes,S. anginosus)***	**3**	**Total no. isolates with sensitivities**	_	2	_	3	3	_	_	_	_	_	_	_
			**No. of susceptible isolates**	_	2	_	3	3	_	_	_	_	_		
			**% Susceptible Isolates**	_	**100%**	_	**100%**	**100%**	_	_	_	_	_		

AST= antimicrobial susceptibility testing; spp.= species; No.= number; MRSA= methicillin resistant *Staphylococcus aureus*; VRE= vancomycin resistant *Enterococcus*

Supplementary Table 7: Gram- negative AST results for adults

Serial number	Organisms	Number of isolates (duplicates removed)	Breakdown of each isolate	% Susceptibility to Routinely Tested Antimicrobial Agents													% Resistance			
				Beta Lactams										Aminoglycosides						
				Ampicillin	Augmentin	Pip-taz	Ceftiaxone	Ceftazidime	Cefepime	Ertapenem	Meropenem	Imipenem	Ciprofloxacin	Getamicin	Amikacin	Cotrimoxazole	% ESBL	% CRE	% MDR	% XDR
1	***Escherichia coli***	**223**	**Total no. isolates with sensitivities**	211	209	218	220	214	221	223	219	221	183	214	210	221	**18%**	**1%**	_	_
			**No. of susceptible isolates**	41	136	198	178	184	197	221	218	219	141	179	206	66				
			**% Susceptible Isolates**	**19%**	**65%**	**91%**	**81%**	**86%**	**89%**	**99%**	**99%**	**99%**	**77%**	**84%**	**98%**	**30%**				
2	***Klebsiella* spp.**	**80**	**Total no. isolates with sensitivities**	_	76	79	78	75	80	73	81	79	59	74	75	80	**28%**	**10%**	_	_
			**No. of susceptible isolates**	_	44	61	49	46	50	70	73	71	41	52	75	47				
			**% Susceptible Isolates**	_	**58%**	**77%**	**63%**	**61%**	**63%**	**96%***	**90%**	**90%**	**69%**	**70%**	**100%**	**59%**				
3	***Salmonella* spp.**	**74**	**Total no. isolates with sensitivities**	69	_	_	72	63	_	71	_	_	69	_	_	73	**2%**	**0%**	_	_
			**No. of susceptible isolates**	58	_	_	71	62	_	71	_	_	58	_	_	61				
			**% Susceptible Isolates**	**84%**	_	_	**99%**	**98%**	_	**100%**	_	_	**84%**	_	_	**84%**				
4	***Pseudomonas* spp.**	**19**	**Total no. isolates with sensitivities**	_	_	18	_	19	18	_	19	19	18	18	19	_	_	_	**0%**	**5%**
			**No. of susceptible isolates**	_	_	16	_	16	16	_	17	17	17	17	18	_				
			**% Susceptible Isolates**	_	_	**89%**	_	**84%**	**89%**	_	**90%**	**90%**	**94%**	**94%**	**95%**	_				
5	***Enterobacter* spp.**	**12**	**Total no. isolates with sensitivities**	_	_	10	12	9	12	9	12	12	8	12	11	11	**8%**	**8%**	_	_
			**No. of susceptible isolates**	_	_	8	10	8	11	9	11	11	7	11	11	8				
			**% Susceptible Isolates**	_	_	**80%**	**83%**	**89%**	**92%**	**100%***	**92%**	**92%**	**88%**	**92%**	**100%**	**73%**				
6	***Acinetobacter* spp.**	**10**	**Total no. isolates with sensitivities**	_	_	10	_	9	8	_	10	9	7	9	6	5	_	_	**10%**	**20%**
			**No. of susceptible isolates**	_	_	7	_	6	5	_	7	7	5	7	6	5				
			**% Susceptible Isolates**	_	_	**70%**	_	**67%**	**63%**	_	**70%**	**78%**	**71%**	**78%**	**100%**	**100%**				

* fewer susceptibility results were available for ertapenem, hence the lowest susceptibility result for a carbapenem was used to determine the CRE rate.

AST=antimicrobial susceptibility testing; spp.= species; No.= number; Pip-taz= piperacillin-tazobactam; ESBL= extended spectrum beta lactamase; CRE= carbapenem resistant Enterobacterales; MDR= multidrug resistant; XDR= extensively drug resistant

Supplementary Table 7 continued: Gram- negative AST results for adults

Serial number	Organisms	Number of isolates (duplicates removed)	Breakdown of each isolate	% Susceptibility to Routinely Tested Antimicrobial Agents													% Resistance			
				Beta Lactams										Aminoglycosides						
				Ampicillin	Augmentin	Pip-taz	Ceftiaxone	Ceftazidime	Cefepime	Ertapenem	Meropenem	Imipenem	Ciprofloxacin	Getamicin	Amikacin	Cotrimoxazole	% ESBL	% CRE	% MDR	% XDR
**7**	***Proteus* spp.**	**12**	**Total no. isolates with sensitivities**	11	10	12	12	11	12	12	12	4	10	12	12	12	**0%**	**0%**	_	_
			**No. of susceptible isolates**	5	9	11	12	11	12	12	12	4	10	12	12	5				
			**% Susceptible Isolates**	**45%**	**90%**	**92%**	**100%**	**100%**	**100%**	**100%**	**100%**	**100%**	**100%**	**100%**	**100%**	**42%**				
**8**	***Haemophilus* spp.**	**7**	**Total no. isolates with sensitivities**	7	0	0	5	_	0	0	5	_	0	_	_	4	_	_	_	_
			**No. of susceptible isolates**	5	_	_	5	_	_	_	5	_	_	_	_	3				
			**% Susceptible Isolates**	**71%**	_	_	**100%**	_	_	_	**100%**	_	_	_	_	**75%**				
**9**	***Salmonella typhi* and *S. paratyphi***	**4**	**Total no. isolates with sensitivities**	4	_	_	4	4	_	4	_	_	3	_	_	4	**0%**	**0%**	_	_
			**No. of susceptible isolates**	2	_	_	4	4	_	4	_	_	2	_	_	2				
			**% Susceptible Isolates**	**50%**	_	_	**100%**	**100%**	_	**100%**	_	_	**67%**	_	_	**50%**				
**10**	***Morganella* spp.**	**4**	**Total no. isolates with sensitivities**	4	3	4	4	4	4	4	4	3	4	4	4	4	**0%**	**0%**	_	_
			**No. of susceptible isolates**	0	0	4	4	4	4	4	4	3	2	2	4	2				
			**% Susceptible Isolates**	**0%**	**0%**	**100%**	**100%**	**100%**	**100%**	**100%**	**100%**	**100%**	**50%**	**50%**	**100%**	**50%**				
**11**	***Serratia marcescens***	**4**	**Total no. isolates with sensitivities**	_	4	0	3	3	4	4	4	0	3	3	3	4	**0%**	**0%**	_	_
			**No. of susceptible isolates**	_	0	_	3	3	4	4	4	_	3	3	3	4				
			**% Susceptible Isolates**	_	**0%**	_	**100%**	**100%**	**100%**	**100%**	**100%**	_	**100%**	**100%**	**100%**	**100%**				
**12**	***Other Enterobacterales (Citrobacter spp.,Providentia rettgeri, Plesiomonas shigelloides,Shigella sonnei)***	**5**	**Total no. isolates with sensitivities**	5	4	4	4	5	4	5	4	3	5	4	4	5	**0%**	**0%**	_	_
			**No. of susceptible isolates**	1	2	4	4	5	4	5	4	3	5	3	3	5				
			**% Susceptible Isolates**		**50%**	**100%**	**100%**	**100%**	**100%**	**100%**	**100%**	**100%**	**100%**	**75%**	**75%**	**100%**				

AST=antimicrobial susceptibility testing; spp.= species; No.= number; Pip-taz= piperacillin-tazobactam; ESBL= extended spectrum beta lactamase; CRE= carbapenem resistant Enterobacterales; MDR= multidrug resistant; XDR= extensively drug resistant

Supplementary Table 7 continued: Gram- negative AST results for adults

Serial number	Organisms	Number of isolates (duplicates removed)	Breakdown of each isolate	% Susceptibility to Routinely Tested Antimicrobial Agents													% Resistance			
				Beta Lactams										Aminoglycosides						
				Ampicillin	Augmentin	Pip-taz	Ceftiaxone	Ceftazidime	Cefepime	Ertapenem	Meropenem	Imipenem	Ciprofloxacin	Getamicin	Amikacin	Cotrimoxazole	% ESBL	% CRE	% MDR	% XDR
13	***Aeromonas* spp.**	**1**	**Total no. isolates with sensitivities**	_	1	0	1	1	1	0	0	1	1	1	1	1	_	_	_	_
			**No. of susceptible isolates**	_	0	_	1	1	1	_	_	0	1	1	1	1				
			**% Susceptible Isolates**	_	**0%**	_	**100%**	**100%**	**100%**	_	_	**0%**	**100%**	**100%**	**100%**	**100%**				
14	***Burkholderia cepacia***	**1**	**Total no. isolates with sensitivities**	_	_	_	_	1	_	_	1	_	_	_	_	1	_	_	_	_
			**No. of susceptible isolates**	_	_	_	_	1	_	_	1	_	_	_	_	0				
			**% Susceptible Isolates**	_	_	_	_	**100%**	_	_	**100%**	_	_	_	_	**0%**				
15	***Stenotrophomonas maltophilia***	**1**	**Total no. isolates with sensitivities**	_	_	_	_	1	_	_	_	_	1	_	_	1	_	_	_	_
			**No. of susceptible isolates**	_	_	_	_	1	_	_	_	_	1	_	_	1				
			**% Susceptible Isolates**	_	_	_	_	**100%**	_	_	_	_	**100%**	_	_	**100%**				
16	***Alcaligenes* spp.**	**1**	**Total no. isolates with sensitivities**	_	_	1	_	1	1	_	1	1	1	1	1	1	_	_	**0%**	**100%**
			**No. of susceptible isolates**	_	_	0	_	0	0	_	0	0	0	0	0	0				
			**% Susceptible Isolates**	_	_	**0%**	_	**0%**	**0%**	_	**0%**	**0%**	**0%**	**0%**	**0%**	**0%**				

AST=antimicrobial susceptibility testing; spp.= species; No.= number; Pip-taz= piperacillin-tazobactam; ESBL= extended spectrum beta lactamase; CRE= carbapenem resistant Enterobacterales; MDR= multidrug resistant; XDR= extensively drug resistant

Supplementary Table 8: Gram- positive AST results for adults

Serial number	Organisms	Number of isolates (duplicates removed)	Breakdown of each isolate	% Susceptibility to Routinely Tested Antimicrobial Agents										Resistance	
				Cloxacillin	Penicillin/ Ampicillin	Clindamycin	Vancomycin	Linezolid	Cotrimoxazole	Rifampicin	Gentamicin high	Ciprofloxacin	Ceftriaxone	% MRSA	% VRE
1	***Streptococcus pneumoniae***	**175**	**Total no. isolates with sensitivities**	_	43	_	171	159	_	_	_		34	_	_
			**No. of susceptible isolates**	_	42	_	171	158	_	_	_		34		
			**% Susceptible Isolates**	_	**98%**	_	**100%**	**99%**	_	_	_		**100%**		
2	***Staphylococcus aureus***	**152**	**Total no. isolates with sensitivities**	148	_	150	150	146	151	148	_	145	_	**7%**	_
			**No. of susceptible isolates**	137	_	133	149	145	94	142	_	131	_		
			**% Susceptible Isolates**	**93%**	_	**89%**	**99%**	**99%**	**62%**	**96%**	_	**90%**	_		
3	***Enterococcus* spp.**	**25**	**Total no. isolates with sensitivities**	_	24	_	23	25	_	_	20	_	_	_	**0%**
			**No. of susceptible isolates**	_	20	_	23	25	_	_	14		_		
			**% Susceptible Isolates**	_	**83%**	_	**100%**	**100%**	_	_	**70%**	_	_		
4	***Streptococcus anginosus***	**22**	**Total no. isolates with sensitivities**	_	21	_	22	20	_	_	_	_	16	_	_
			**No. of susceptible isolates**	_	21	_	22	20	_	_	_	_	16		
			**% Susceptible Isolates**	_	**100%**	_	**100%**	**100%**	_	_	_	_	**100%**		
5	***S.pyogenes***	**18**	**Total no. isolates with sensitivities**	_	17	_	17	18	_	_	_	_	16	_	_
			**No. of susceptible isolates**	_	17	_	17	18	_	_	_	_	16		
			**% Susceptible Isolates**	_	**100%**	_	**100%**	**100%**	_	_	_	_	**100%**		
6	***S.agalactiae***	**12**	**Total no. isolates with sensitivities**	_	12	_	11	11	_	_	_		3	_	_
			**No. of susceptible isolates**	_	12	_	11	11	_	_	_	_	3		
			**% Susceptible Isolates**	_	**100%**	_	**100%**	**100%**	_	_	_	_	**100%**		
7	***Other Streptococci (S. dysgalactiae, S.gallolyticus, Gemella spp., Veilonella spp.***	**9**	**Total no. isolates with sensitivities**	_	9	_	8	8	_	_	_	_	8	_	_
			**No. of susceptible isolates**	_	9	_	8	8	_	_	_	_	8		
			**% Susceptible Isolates**	_	**100%**	_	**100%**	**100%**	_	_	_	_	**100%**		
8	***S. lugdunensis***	**1**	**Total no. isolates with sensitivities**	1	_	1	1	1	1	1	_	1	_	_	_
			**No. of susceptible isolates**	1	_	0	1	1	1	1	_	1	_		
			**% Susceptible Isolates**	**100%**	_	**0%**	**100%**	**100%**	**100%**	**100%**	_	**100%**	_		

AST= antimicrobial susceptibility testing; spp.= species; No.= number; MRSA= methicillin resistant *Staphylococcus aureus*; VRE= vancomycin resistant *Enterococcus*

Supplementary Table 9: Gram- negative AST results for the elderly

Serial number	Organisms	Number of isolates (duplicates removed)	Breakdown of each isolate	% Susceptibility to Routinely Tested Antimicrobial Agents													% Resistance			
				Beta Lactams										Aminoglycosides						
				Ampicillin	Augmentin	Pip-taz	Ceftiaxone	Ceftazidime	Cefepime	Ertapenem	Meropenem	Imipenem	Ciprofloxacin	Getamicin	Amikacin	Cotrimoxazole	% ESBL	% CRE	% MDR	% XDR
1	***Escherichia coli***	**61**	**Total no. isolates with sensitivities**	55	59	61	58	53	61	59	59	57	50	58	57	61	**15%**	**2%**	_	_
			**No. of susceptible isolates**	11	39	52	49	48	56	59	58	56	36	51	55	21				
			**% Susceptible Isolates**	**20%**	**66%**	**85%**	**84%**	**91%**	**92%**	**100%***	**98%**	**98%**	**72%**	**88%**	**96%**	**34%**				
2.	***Klebsiella spp.***	**28**	**Total no. isolates with sensitivities**	_	28	27	26	28	28	27	28	28	24	26	27	27	**46%**	**18%**	_	_
			**No. of susceptible isolates**	_	16	19	14	16	17	23	23	23	16	16	27	13				
			**% Susceptible Isolates**	_	**57%**	**70%**	**54%**	**57%**	**61%**	**85%***	**82%**	**82%**	**67%**	**62%**	**100%**	**48%**				
3	***Enterobacter* spp.**	**13**	**Total no. isolates with sensitivities**	_	_	11	13	13	11	11	13	12	11	12	13	13	**31%**	**0%**	_	_
			**No. of susceptible isolates**	_	_	9	9	9	9	11	13	12	9	9	12	9				
			**% Susceptible Isolates**	_	_	**82%**	**69%**	**69%**	**82%**	**100%**	**100%**	**100%**	**82%**	**75%**	**92%**	**69%**				
4	***Proteus* spp.**	**10**	**Total no. isolates with sensitivities**	10	10	9	10	10	9	9	9	2	8	10	10	10	**0%**	**0%**	_	_
			**No. of susceptible isolates**	4	6	8	10	10	9	9	9	2	7	10	9	8				
			**% Susceptible Isolates**	**40%**	**60%**	**89%**	**100%**	**100%**	**100%**	**100%**	**100%**	**100%**	**88%**	**100%**	**90%**	**80%**				
5	***Salmonella* spp.**	**9**	**Total no. isolates with sensitivities**	9	_	_	9	9	_	9	_	_	8	_	_	9	**0%**	**0%**	_	_
			**No. of susceptible isolates**	8	_	_	9	9	_	9	_	_	7	_	_	8				
			**% Susceptible Isolates**	**89%**	_	_	**100%**	**100%**	_	**100%**	_	_	**88%**			**89%**				
6	***Pseudomonas* spp.**	**8**	**Total no. isolates with sensitivities**	_	_	7	_	8	8	_	8	8	7	8	8	_	_	_	**0%**	**25%**
			**No. of susceptible isolates**	_	_	6	_	5	6	_	6	6	5	5	5	_				
			**% Susceptible Isolates**	_	_	**85%**	_	**63%**	**75%**	_	**75%**	**75%**	**71%**	**63%**	**63%**	_				

*fewer susceptibility results were available for ertapenem, hence the lowest susceptibility result for a carbapenem was used to determine the CRE rate.

AST=antimicrobial susceptibility testing; spp.= species; No.= number; Pip-taz= piperacillin-tazobactam; ESBL= extended spectrum beta lactamase; CRE= carbapenem resistant Enterobacterales; MDR= multidrug resistant; XDR= extensively drug resistant

Supplementary Table 9 continued : Gram- negative AST results for the elderly

Serial number	Organisms	Number of isolates (duplicates removed)	Breakdown of each isolate	% Susceptibility to Routinely Tested Antimicrobial Agents													% Resistance			
				Beta Lactams										Aminoglycosides						
				Ampicillin	Augmentin	Pip-taz	Ceftiaxone	Ceftazidime	Cefepime	Ertapenem	Meropenem	Imipenem	Ciprofloxacin	Getamicin	Amikacin	Cotrimoxazole	% ESBL	% CRE	% MDR	% XDR
7	***Haemophilus influenzae***	**3**	**Total no. isolates with sensitivities**	3	0	0	3	_	0	_	3	_	0	_	_	3				
			**No. of susceptible isolates**	1	_	_	2	_	_	_	3	_	_	_	_	2	_	_	_	_
			**% Susceptible Isolates**	**33%**	_	_	**67%**	_	_	_	**100%**	_	_	_	_	**67%**				
8	***Acinetobacter* spp.**	**2**	**Total no. isolates with sensitivities**	_	_	2	_	2	2	_	2	2	1	1	2	1				
			**No. of susceptible isolates**	_	_	1	_	0	0	_	1	1	1	1	2	0	_	_	**0%**	**0%**
			**% Susceptible Isolates**	_	_	**50%**	_	**0%**	**0%**	_	**50%**	**50%**	**100%**	**100%**	**100%**	**0%**				
9	***Other Enterobacterales (Serratia* spp., *Morganella* spp.).**	**3**	**Total no. isolates with sensitivities**	_	_	3	3	3	3	3	3	2	2	3	3	3				
			**No. of susceptible isolates**	_	_	3	3	3	3	3	3	2	2	2	3	2	**0%**	**0%**	_	_
			**% Susceptible Isolates**	_	_	**100%**	**100%**	**100%**	**100%**	**100%**	**100%**	**100%**	**100%**	**67%**	**100%**	**67%**				
10	***Aeromonas* spp.**	**1**	**Total no. isolates with sensitivities**	_	1	1	1	1	1	0	0	1	0	1	1	1				
			**No. of susceptible isolates**	_	1	1	1	1	1	_	_	1	_	1	1	1	_	_	_	_
			**% Susceptible Isolates**	_	**100%**	**100%**	**100%**	**100%**	**100%**	_	_	**100%**	_	**100%**	**100%**	**100%**				
11	***Burkholderia cepacia***	**1**	**Total no. isolates with sensitivities**	_	_	_	_	1	_	_	1	_	_	_	_	1				
			**No. of susceptible isolates**	_	_	_	_	1	_	_	1	_	_	_	_	1	_	_	_	_
			**% Susceptible Isolates**	_	_	_	_	**100%**	_	_	**100%**	_	_	_	_	**100%**				

AST=antimicrobial susceptibility testing; spp.= species; No.= number; Pip-taz= piperacillin-tazobactam; ESBL= extended spectrum beta lactamase; CRE= carbapenem resistant Enterobacterales; MDR= multidrug resistant; XDR= extensively drug resistant

Supplementary Table 10: Gram- positive AST results for the elderly

Serial number	Organisms	Number of isolates (duplicates removed)	Breakdown of each isolate	% Susceptibility to Routinely Tested Antimicrobial Agents										Resistance	
				Cloxacillin	Penicillin/ Ampicillin	Clindamycin	Vancomycin	Linezolid	Cotrimoxazole	Rifampicin	Gentamicin higl	Ciprofloxacin	Ceftriaxone	% MRSA	% VRE
**1**	***Staphylococcus aureus***	**26**	**Total no. isolates with sensitivities**	26	_	26	26	25	25	26	_	24	_	**15%**	_
			**No. of susceptible isolates**	22	_	22	26	25	14	24	_	19	_		
			**% Susceptible Isolates**	**85%**	_	**85%**	**100%**	**100%**	**56%**	**92%**	_	**79%**	_		
**2**	***Streptococcus pneumoni***	**12**	**Total no. isolates with sensitivities**	_	4	_	11	10	_	_	_	_	3	_	_
			**No. of susceptible isolates**	_	4	_	11	10	_	_	_	_	3		
			**% Susceptible Isolates**	_	**100%**	_	**100%**	**100%**	_	_	_	_	**100%**		
**3**	***Enterococcus* spp.**	**8**	**Total no. isolates with sensitivities**	_	7	_	8	7	_	_	6	_	_	_	**0%**
			**No. of susceptible isolates**	_	5	_	8	7	_	_	4	_	_		
			**% Susceptible Isolates**	_	**71%**	_	**100%**	**100%**	_	_	**67%**	_	_		
**4**	***Streptococcus pyogenes***	**4**	**Total no. isolates with sensitivities**	_	4	_	3	4	_	_	_	_	3	_	_
			**No. of susceptible isolates**	_	4	_	3	4	_	_	_	_	3		
			**% Susceptible Isolates**	_	**100%**	_	**100%**	**100%**	_	_	_	_	**100%**		
**5**	***Streptococcus anginosus***	**4**	**Total no. isolates with sensitivities**	_	4	_	3	3	_	_	_	_	3	_	_
			**No. of susceptible isolates**	_	4	_	3	3	_	_	_	_	3		
			**% Susceptible Isolates**	_	**100%**	_	**100%**	**100%**	_	_	_	_	**100%**		
**6**	***Streptococcus agalactiae***	**3**	**Total no. isolates with sensitivities**	_	3	_	2	3	_	_	_	_	2	_	_
			**No. of susceptible isolates**	_	3	_	2	3	_	_	_	_	2		
			**% Susceptible Isolates**	_	**100%**	_	**100%**	**100%**	_	_	_	_	**100%**		
**7**	***Staphylococcus lugdunensis***	**1**	**Total no. isolates with sensitivities**	_	1	_	1	1	_	_	_	_	_	_	_
			**No. of susceptible isolates**	_	0	_	1	1	_	_	_	_	_		
			**% Susceptible Isolates**	_	**0%**	_	**100%**	**100%**	_	_	_	_	_		
**8**	***Other Streptococci (S. dysgalactiae, Leuconostoc* spp.)**	**2**	**Total no. isolates with sensitivities**	_	1	_	2	2	_	_	_	_	2	_	_
			**No. of susceptible isolates**	_	1	_	1	2	_	_	_	_	2		
			**% Susceptible Isolates**	_	**100%**	_	**50%**	**100%**	_	_	_	_	**100%**		

AST= antimicrobial susceptibility testing; spp.= species; No.= number; MRSA= methicillin resistant *Staphylococcus aureus*; VRE= vancomycin resistant *Enterococcus*

Supplementary Table 11: Gram- negative AST results for surgical ED

Serial number	Organisms	Number of isolates (duplicates removed)	Breakdown of each isolate	% Susceptibility to Routinely Tested Antimicrobial Agents													% Resistance			
				Beta Lactams										Aminoglycosides						
				Ampicillin	Augmentin	Pip-taz	Ceftiaxone	Ceftazidime	Cefepime	Ertapenem	Meropenem	Imipenem	Ciprofloxacin	Getamicin	Amikacin	Cotrimoxazole	% ESBL	% CRE	% MDR	% XDR
1	***Klebsiella pneumoniae***	7	Total no. isolates with sensitivities	_	6	7	7	7	7	5	7	7	5	6	6	7	29%	0%	_	_
			No. of susceptible isolates	_	3	7	5	5	5	5	7	7	5	4	6	4				
			% Susceptible Isolates	_	50%	100%	71%	71%	71%	100%	100%	100%	100%	67%	100%	57%				
2	***Acinetobacter* spp.**	7	Total no. isolates with sensitivities	_	_	7	_	6	5	_	6	6	5	7	5	7	_	_	57%	14%
			No. of susceptible isolates	_	_	2	_	2	2	_	2	2	5	7	4	2				
			% Susceptible Isolates	_	_	29%	_	33%	40%	_	33%	33%	100%	100%	80%	29%				
3	***Escherichia coli***	3	Total no. isolates with sensitivities	3	3	3	3	3	3	3	3	3	3	3	3	3	33%	0%	_	_
			No. of susceptible isolates	1	2	2	2	3	3	3	3	3	2	2	3	1				
			% Susceptible Isolates	33%	67%	67%	67%	100%	100%	100%	100%	100%	67%	67%	100%	33%				
4	***Proteus* spp.**	3	Total no. isolates with sensitivities	3	3	3	2	3	3	2	3	0	2	3	3	3	33%	0%	_	_
			No. of susceptible isolates	1	3	3	2	2	2	2	3	_	1	2	2	2				
			% Susceptible Isolates	33%	100%	100%	100%	67%	67%	100%	100%	_	50%	67%	67%	67%				
5	***Salmonella* spp.**	2	Total no. isolates with sensitivities	2	_	_	1	0	_	2	_	_	2	_	_	2	0%	0%	_	_
			No. of susceptible isolates	2	_	_	1	_	_	2	_	_	2	_	_	2				
			% Susceptible Isolates	100%	_	_	100%	_	_	100%	_	_	100%	_	_	100%				
6	***Serratia* spp.**	2	Total no. isolates with sensitivities	_	2	1	1	1	1	1	2	1	1	1	1	1	0%	50%	_	_
			No. of susceptible isolates	_	0	1	1	0	1	0	1	1	1	1	1	0				
			% Susceptible Isolates	_	0%	100%	100%	0%	100%	0%*	50%	100%	100%	100%	100%	0%				
7	***Enterobacter* spp.**	1	Total no. isolates with sensitivities	_	_	1	1	1	0	1	1	1	1	1	1	1	0%	0%	_	_
			No. of susceptible isolates	_	_	1	1	1	_	1	1	1	1	1	1	1				
			% Susceptible Isolates	_	_	100%	100%	100%	_	100%	100%	100%	100%	100%	100%	100%				
8	***Haemophilus* spp.**	1	Total no. isolates with sensitivities	1	0	0	0	_	0	0	1	_	1	_	_	1	_	_	_	_
			No. of susceptible isolates	1	_	_	_	_	_	_	1	_	1	_	_	1				
			% Susceptible Isolates	100%	_	_	_	_	_	_	100%	_	100%	_	_	100%				

*fewer susceptibility results were available for ertapenem, hence susceptibility to meropenem was used to determine the CRE rate.

Supplementary Table 12: Gram- positive AST results for surgical ED

Serial number	Organisms	Number of isolates (duplicates removed)	Breakdown of each isolate	% Susceptibility to Routinely Tested Antimicrobial Agents											Resistance	
				Cloxacillin	Penicillin/ Ampicillin	Erythromycin	Clindamycin	Vancomycin	Linezolid	Cotrimoxazole	Rifampicin	Gentamicin	Gentamicin hig[illegible]	Ciprofloxacin	% MRSA	% VRE
**1**	***Staphylococcus aureus***	**8**	**Total no. isolates with sensitivities**	8	_	7	7	8	8	8	8	8	6	6	_	_
			**No. of susceptible isolates**	8	_	7	7	8	8	4	8	8	6	6		
			**% Susceptible Isolates**	**100%**		**100%**	**100%**	**100%**	**100%**	**50%**	**100%**	**100%**	**100%**	**100%**		
**2**	***Streptococcus pneumoniae***	**6**	**Total no. isolates with sensitivities**	_	3	_	_	6	5	_	_	_	_	_	_	_
			**No. of susceptible isolates**	_	3	_	_	6	5	_	_	_	_	_		
			**% Susceptible Isolates**	_	**100%**	_	_	**100%**	**100%**	_	_	_	_	_		
**3**	***Enterococcus faecalis***	**2**	**Total no. isolates with sensitivities**	_	2	_	_	2	2	_	_	_	_	_	0%	0%
			**No. of susceptible isolates**	_	2	_	_	2	2	_	_	_	_	_		
			**% Susceptible Isolates**	_	**100%**	_	_	**100%**	**100%**	_	_	_	_	_		
**4**	***Enterococcus faecium***	**1**	**Total no. isolates with sensitivities**	_	1	_	_	1	1	_	_	_	_	_	0%	0%
			**No. of susceptible isolates**	_	0	_	_	1	1	_	_	_	_	_		
			**% Susceptible Isolates**	_	**0,0%**	_	_	**100%**	**100%**	_	_	_	_	_		
**5**	***Enterococcus* spp.**	**1**	**Total no. isolates with sensitivities**	_	1	_	_	1	1	_	_	_	_	_	0%	0%
			**No. of susceptible isolates**	_	1	_	_	1	1	_	_	_	_	_		
			**% Susceptible Isolates**	_	**100%**	_	_	**100%**	**100%**	_	_	_	_	_		

AST= antimicrobial susceptibility testing; spp.= species; No.= number; MRSA= methicillin resistant *Staphylococcus aureus*; VRE= vancomycin resistant *Enterococcus*

Supplementary Table 13: *Candida* species AST results from paediatric ED

Serial number	Organisms	Number of isolates (duplicates removed)	% of total *Candida* spp.	Breakdown of each isolate	% Susceptibility to Routinely Tested Azoles	
					Fluconazole	Voriconazole
**Neonates**						
1	*Candida parapsilosis*	1	100%	Total no. isolates with sensitivities	1	0
				No. of susceptible isolates	0	_
				% Susceptible Isolates	0%	_
**Infants**						
2	*Candida albicans*	1	50%	Total no. isolates with sensitivities	0	0
				No. of susceptible isolates	_	_
				% Susceptible Isolates	_	_
3	*Candida lusitaniae*	1	50%	Total no. isolates with sensitivities	0	0
				No. of susceptible isolates	_	_
				% Susceptible Isolates	_	_
**Older Children**						
4	*Candida albicans*	1	100%	Total no. isolates with sensitivities	1	1
				No. of susceptible isolates	1	1
				% Susceptible Isolates	100%	100%

Supplementary Table 14: *Candida* species AST results for adults and elderly

Serial number	Organisms	Number of isolates (duplicates removed)	% of total Candida spp.	Breakdown of each isolate	% Susceptibility to Routinely Tested Azoles: Fluconazole	% Susceptibility to Routinely Tested Azoles: Voriconazole
**Adults**						
1	Candida albicans	6	55%	Total no. isolates with sensitivities	5	6
				No. of susceptible isolates	5	6
				% Susceptible Isolates	100%	100%
2	Candida parapsilosis	2	18%	Total no. isolates with sensitivities	2	1
				No. of susceptible isolates	2	1
				% Susceptible Isolates	100%	100%
3	Candida glabrata	1	9%	Total no. isolates with sensitivities	0	0
				No. of susceptible isolates	NIL CLSI BREAKPOINTS	
				% Susceptible Isolates	_	_
4	Candida tropicalis	1	9%	Total no. isolates with sensitivities	1	1
				No. of susceptible isolates	1	1
				% Susceptible Isolates	100%	100%
5	Candida spp.	1	9%	Total no. isolates with sensitivities	0	0
				No. of susceptible isolates	_	_
				% Susceptible Isolates	_	_
**Elderly**						
6	Candida albicans	1	100%	Total no. isolates with sensitivities	1	1
				No. of susceptible isolates	1	1
				% Susceptible Isolates	100%	100%

Supplementary Table 15: *Candida* species AST results for surgical ED

Surgical ED						
**Serial number**	**Organism**	**Number of isolates (duplicates removed)**	**%of total *Candida* spp.**	**Breakdown of each isolate**	**Fluconazole**	**Voriconazole**
1	*Candida glabrata*	1	100%	Total no. isolates with sensitivities	1	1
				No. of susceptible isolates	No CLSI breakpoints	
				% Susceptible Isolates	–	–
